# Management of Advanced Peri-Implantitis by Guided Bone Regeneration in Combination with Trabecular Metal Fixtures, Two Months after Removal of the Failed Implants: Two-Year Results of a Single-Cohort Clinical Study

**DOI:** 10.3390/jcm13030713

**Published:** 2024-01-25

**Authors:** Stefano Bianconi, Georgios Romanos, Tiziano Testori, Massimo Del Fabbro

**Affiliations:** 1 Department of Oral Surgery and Dentistry, General Hospital, 39100 Bolzano, Italy; bianconistefano@gmail.com; 2 Department of Periodontics and Endodontics, Stony Brook University, Stony Brook, NY 11794, USA; georgios.romanos@stonybrookmedicine.edu; 3 Department of Biomedical, Surgical and Dental Sciences, Università degli Studi di Milano, 20122 Milan, Italy; info@tiziano-testori.it; 4 Department of Implantology and Oral Rehabilitation, Dental Clinic, IRCCS Ospedale Galeazzi-Sant’Ambrogio, 20157 Milan, Italy; 5 Department of Periodontics and Oral Medicine, School of Dentistry, University of Michigan, Ann Arbor, MI 48109, USA; 6 Department of Oral Medicine, Infection and Immunity, Harvard School of Dental Medicine, Boston, MA 01451, USA; 7 Fondazione IRCCS Cà Granda, Ospedale Maggiore Policlinico, 20122 Milan, Italy

**Keywords:** dental implants, guided bone regeneration, implant explantation, implant stability, osseointegration, peri-implantitis, surgical treatment

## Abstract

**Background:** Implant replacement is among the treatment options for severe peri-implantitis. The aim of this single-cohort study was to evaluate the feasibility of replacing compromised implants affected by advanced peri-implantitis with new implants with a porous trabecular metal (TM) structure. **Materials and Methods:** Patients with one or more implants in the posterior region showing a defect depth >50% of implant length, measured from the residual crest, were consecutively included. Two months after implant removal, patients received a TM implant combined with a xenograft and a resorbable membrane. The implant stability quotient (ISQ) was measured at placement and re-assessed five months later (at uncovering), then after 6, 12, and 24 months of function. Marginal bone loss was radiographically evaluated. **Results:** Twenty consecutive cases were included. One patient dropped out due to COVID-19 infection, and nineteen cases were evaluated up to 24 months. At placement, the mean ISQ was 53.08 ± 13.65 (standard deviation), which increased significantly to 69.74 ± 9.01 after five months of healing (*p* < 0.001) and to 78.00 ± 7.29 after six months of loading (*p* < 0.001). Thereafter, the ISQ remained stable for up to 24 months (80.55 ± 4.73). All implants successfully osseointegrated and were restored as planned. After two years, the average marginal bone level change was −0.41 ± 0.38 mm (95% confidence interval −0.60, −0.21), which was limited yet significantly different from the baseline (*p* < 0.05). **Conclusions:** The treatment of advanced peri-implant defects using TM implants inserted two months after explantation in combination with guided bone regeneration may achieve successful outcomes up to two years follow-up, even in the presence of low primary stability.

## 1. Introduction

Pathological processes affecting the tissues around osseointegrated dental implants have gained increased attention in the dental community in recent years. The term “peri-implantitis” was introduced decades ago to describe an infectious pathological condition of peri-implant tissues [1]. During the World Workshop on the Classification of Periodontal and Peri-implant Diseases and Conditions of 2017, a new definition for peri-implant health and diseases was introduced [2]. Peri-implantitis is characterized by the non-reversible and progressive loss of supporting bone, which may be observed radiographically, and peri-implant mucosal inflammation [3].

Peri-implantitis occurs primarily as a result of an overwhelming bacterial challenge and subsequent uncontrolled host immune response [4,5]. According to the recent literature, a key role for the onset and progression of peri-implantitis can be ascribed to the oxidative stress due to diabetes mellitus [6] and other unfavorable local and systemic metabolic conditions causing an inflammatory burden [7], in association with a polymicrobial infection that may affect peri-implant bone and soft tissues [8]. Also, like periodontitis, a greater risk of developing peri-implantitis as well as worse outcomes of both surgical and non-surgical treatment have been observed in tobacco smokers, as compared to non-smokers [9]. A recent comparative study demonstrated that smoking cigarettes may adversely affect the primary and secondary stability of implants immediately placed in post-extraction sockets [10]. Several studies highlighted that there are further osteoimmuno-inflammatory mediators, different from those induced by bacteria, that could promote a host immune response leading to the breakdown of peri-implant tissue [11,12,13,14,15,16,17]. In the last decade, it has also been hypothesized that peri-implantitis might simply be a sort of foreign body reaction [18]. In addition, other factors may be recognized as potentially affecting peri-implant tissue health, such as implant mispositioning, excessive masticatory or extra-axial load, cementation residues, emergency of the abutment’s profile, implant–abutment connection gap, mixed tooth/implant prosthetic solutions, a previous history of periodontitis, compliance with supportive therapy, and a lack of periodical radiographic and clinical controls in the follow-up [19].

The prevalence of peri-implant diseases has been reported in several longitudinal and cross-sectional studies [20,21,22,23,24,25,26], as well as in systematic reviews and meta-analyses. These studies estimated a weighted mean prevalence of 43% (range 19 to 65%) for peri-implant mucositis and of 22% (range 1 to 47%) for peri-implantitis [27,28]. The wide variability in prevalence data may depend on the population screened and on the peri-implantitis case definition adopted [28].

Decision making for medical and surgical treatment approaches to peri-implantitis is challenging as no consensus exists among clinicians. In fact, convincing evidence is not available to date, although peri-implantitis treatment is one of the most debated topics among the community of implantologists [29,30,31,32,33].

A range of solutions has been proposed to address peri-implant disease, with their invasiveness related to the severity of the peri-implant bone loss and peri-implant tissue condition [34]. In conditions of lower severity, treatments ranged from site decontamination (debridement, polishing of the implant surface, laser decontamination) to implantoplasty (smoothening of the implant surface to reduce the risk of bacterial adhesion). Treatments for severe conditions ranged from regenerative procedures with autogenous bone and/or bone substitute materials [35,36,37] to the removal of the compromised implant and substitution with another implant subsequent to/in combination with bone regeneration procedures [38,39]. The last situation can be considered analogue to implant placement in post-extraction sockets of a compromised tooth affected by a wide periodontal defect.

After implant removal in advanced peri-implantitis cases, especially the ones showing large crater-like defects, it is often difficult to achieve the primary stability of the new implant due to the lack of hard tissue support. In many cases, a preliminary regenerative procedure, with bone grafting into the defect site and the placement of a barrier membrane, may be necessary to re-create enough hard tissue over several months of healing to provide support and stability for the placement of a new implant. All these procedures, however, may be frustrating for the patients and the clinicians. The possibility of predictably achieving implant osseointegration, even in the absence of optimal stability at placement, would accelerate treatment procedures and recovery in cases of large defects, thus increasing patients’ acceptance of implant therapy.

In previous studies, implants placed two months after tooth removal in wide post-extraction defects, with less-than-optimal primary stability via compaction of a surrounding bone substitute, successfully osseointegrated for functional loading after five months [40,41]. The rationale of such an interval between tooth extraction and implant insertion was mainly to allow for soft tissue healing involving an adequate amount of keratinized tissue around the implant at placement. In addition, such implants could achieve secondary stability after six months of function, similar to implants inserted with good primary stability [42]. The peculiar trabecular structure of the implant used in these investigations might have overcome the issue of lacking primary stability, thus allowing for successful rehabilitation in combination with a reduction in the total treatment time. These studies suggested that in the presence of wide post-extraction defects, the choice of implant design may become crucially important.

The aim of the present study was to evaluate the reliability of treating cases of advanced peri-implantitis, requiring implant removal through a protocol based on bone regeneration and early reinsertion of a new implant with a porous three-dimensional trabecular mid-section structure. The hypothesis was that peri-implantitis can be successfully and predictably treated by replacing compromised implants with a trabecular metal structure, combined with guided bone regeneration, to promote peri-implant defect regeneration.

## 2. Materials and Methods

### 2.1. Study Population

The study protocol was approved by the Regional Ethical Committee of the Regional Hospital of Bolzano, Italy (Protocol n.41398\2017). All included patients were informed of the study protocol and were enrolled only after they signed a consent to participate and a privacy policy form. All patients were enrolled and treated at the General Hospital of Bolzano between September 2019 and April 2020 while following the principles enclosed in the 1980 Helsinki Declaration for biomedical research involving human subjects, as revised in 2013.

### 2.2. Inclusion Criteria

The main inclusion criteria for candidates to implant surgery were as follows: patients of at least 18 years of age, in good general health, and able to undergo surgical and restorative procedures (ASA-1 and ASA-2 according to the American Society of Anesthesiologists classification system). Specific criteria for this study were as follows: patients with at least one implant inserted in the posterior jaws, displaying a pocket deeper than 50% of the implant length [43] both mesially and distally with respect to the crestal level, clinical implant mobility, and suppuration and/or bleeding on probing. Only bone defects classified as 3\4 walls were included. The diagnosis for patient inclusion was based on panoramic and intraoral radiographs and clinical assessment. The defect depth was measured on periapical radiographs, as the distance between the bone crest level and the deepest part of the radiolucency, at both mesial and distal sides. The calibration was based on known implant length.

### 2.3. Exclusion Criteria

The exclusion criteria were as follows: patients taking drugs that influence bone metabolism (e.g., antiresorptive drugs such as bisphosphonates), patients undergoing chemotherapy, patients with uncontrolled systemic conditions (e.g., diabetes), alcohol abuse, heavy smokers (smoking more than 10 cigarettes/day for more than 10 years), patients with parafunction (bruxism or clenching), and uncooperative patients not available to return for each follow-up control visit.

### 2.4. Surgical Protocol

**Figure 1:**
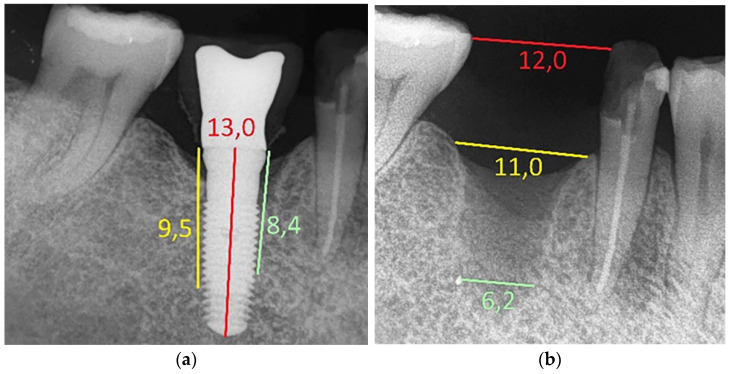
(**a**) Radiograph of a compromised implant positioned in the right first molar region. Although less evident on the X-ray than in case 1, peri-implantitis is more severe. In fact, the depths are 8.4 mm mesially and 9.5 mm distally, measured from the crestal level and calibrated on the actual length of the implant (13 mm). It is probable that the original positioning of this implant was subcrestal. At the time of visit, bleeding on probing and inflammation of the gingiva around the crown were noted. (**b**) After implant removal, the width of the defect is evident. Calibrated on the real distance between the teeth, the size of the bone defect was 11.0 mm mesio-distally at crestal level and 6.2 mm at the middle and apical part.

A single experienced operator (S.B.) performed all surgical procedures. The first step was dedicated to the failing implant removal (Figure 1a) and curettage of the site for all included patients. One hour before surgery, patients received 2 g of amoxicillin and clavulanic acid (or, if allergic, 600 mg of clindamicin) and mouth-rinsed with 0.2% chlorexidine for 2 min. Patients were treated under local anesthesia using articaine 4% and adrenaline 1:100,000. A full-thickness flap was elevated to access the defect. Very careful implant removal was carried out to minimize the traumatic effect on surrounding hard and soft tissues using the counter-torque technique and specific ultrasonic inserts (Piezosurgery^®^, Mectron, Carasco, Genova, Italy). No trephine bur was used for implant removal (Figure 1b). All the granulation tissue was removed to allow for better and faster closure of the soft tissues. After curettage, a collagen sponge was inserted, and soft tissues were allowed to heal by secondary healing. An intraoral radiograph was taken after implant removal. The patients were prescribed with naproxen sodium 550 mg tablets twice daily for pain control if needed and chlorhexidine digluconate mouthwash 0.2% twice daily for 7 days for plaque control.

**Figure 2:**
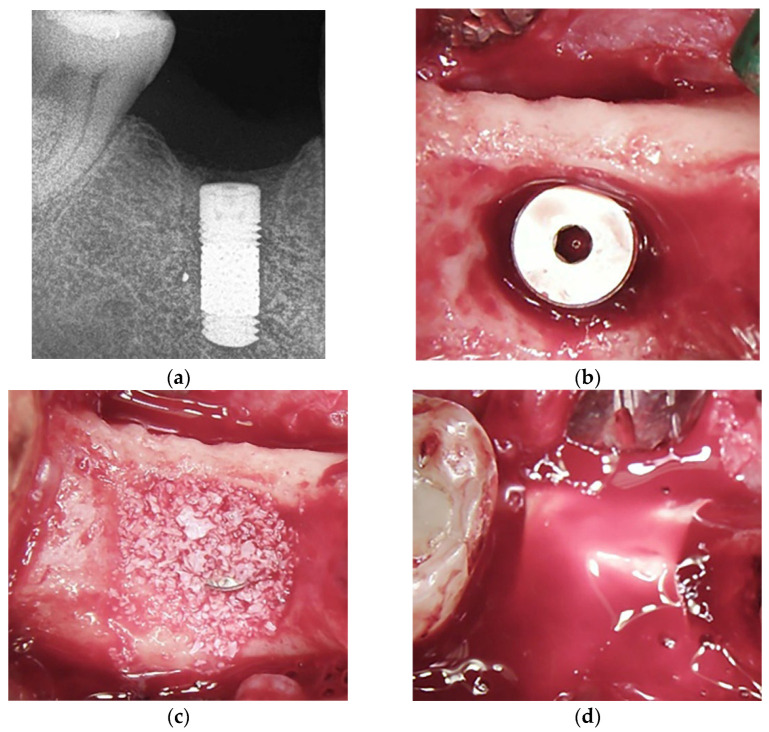
Two months after implant removal. (**a**) Radiograph taken the day of insertion of the TM implant. After TM implant placement, the gap between the implant surface and the bone wall was filled with small particles for augmentation. To keep a distance of at least 1.5 mm from the implant surface to the buccal/lingual outer walls, a 4.7 × 11.5 mm implant was chosen. The ISQ values at placement were 54 and 53 in the vestibulo-lingual and mesio-distal directions, respectively, indicating a low primary stability. (**b**) After implant positioning, the gap all around the implant is visible. We chose to fill the gap only after implant positioning, to allow for the blood to fill the spaces of the trabecular structure, thus promoting the osseoincorporation process. (**c**) The augmentation material consisted of small particles (0.25–1 mm) of porcine bone, which were chosen to completely fill the defect without compression. (**d**) After the placement of a covering pericardium membrane, the flaps were closed without tension using PTFE suture, which was removed after 10 days.

After two months, the patients were called in for the second step consisting of guided bone regeneration (GBR) concomitant with new implant insertion (Figure 2a). A second periapical radiograph was taken before implant placement. One hour before the surgery, the patient received the same prophylactic regimen described before. The procedure was carried out under local anesthesia using articaine 4% and adrenaline 1:100,000. After flap elevation, a thorough and careful curettage of the defect site was performed to remove any residual granular tissue and to induce bleeding before grafting. Trabecular metal (TM) implants with a machined collar were used in the following sizes: 3.7, 4.1, and 4.7 mm diameters and 10, 11.5, and 13 mm lengths (Zimmer Biomet Dental, Palm Beach Gardens, FL, USA). The diameter of the new TM implant was chosen to leave a bone wall thickness of at least 1.5 mm at the buccal and lingual/palatal side (39) (Figure 2b). After implant insertion, a porcine particulate with a granule size of 0.25 to 1 mm was used as bone grafting material (RegenerOss^®^, Zimmer Biomet Dental) to fill the defect around implants until the desired ridge contour was established (Figure 2c). The implant stability quotient (ISQ) was evaluated by means of resonance frequency analysis, as described below. After cover screw placement, the graft and the implant were covered using a pericardium membrane (CopiOs^®^, Zimmer Biomet Dental) (Figure 2d), and the flap was closed with a single PTFE suture (Cytoplast^®^, Osteogenic Biomedical, Lubbock, TX, USA).

As post-operative care, naproxen sodium 550 mg tablets twice daily for pain control if needed and chlorhexidine digluconate mouthwash 0.2% twice daily for 7 days for plaque control were prescribed. One week later, the patient was recalled for suture removal, and an intraoral radiograph was taken.

### 2.5. Follow-Up

**Figure 3:**
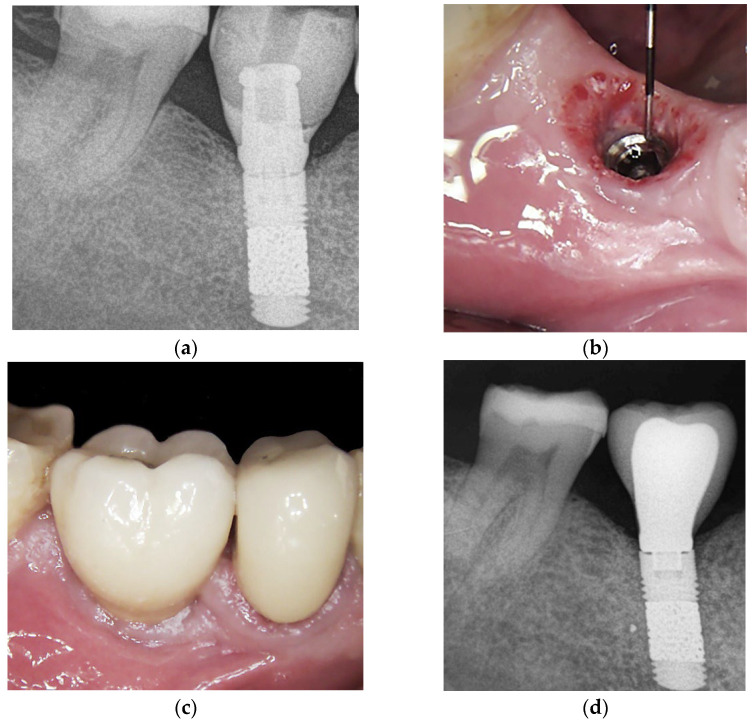
(**a**) Periapical X-ray after 5 months at provisional crown delivery. After 6 months, the provisional crown was removed and the soft tissue was evaluated and the ISQ values were assessed at 74 and 72 in the vestibulo-lingual and mesio-distal directions, respectively (**b**). The final ceramic crown was delivered (**c**); no bone loss was visible (**d**).

The first control was performed after five months of healing, at the prosthetic phase, in which the status of the graft and the implant was evaluated clinically and radiographically. Implants were surgically exposed, the cover screw was removed, and the ISQ value was assessed. Fourteen days later, a screw-retained provisional restoration, made of composite resin (Figure 3a), was delivered for assessment of the ISQ values during the functional loading period. The provisional screw-retained prosthesis allowed for a progressive loading of the restoration, which is necessary for optimal bone trabeculae organization around and within the TM implant under loading. After six months, the provisional screw-retained prosthesis was removed and the peri-implant soft tissue was evaluated (Figure 3b) and substituted by a ceramic crown (Figure 3c), and a periapical X-ray was taken showing stable bone levels (Figure 3d).

**Figure 4:**
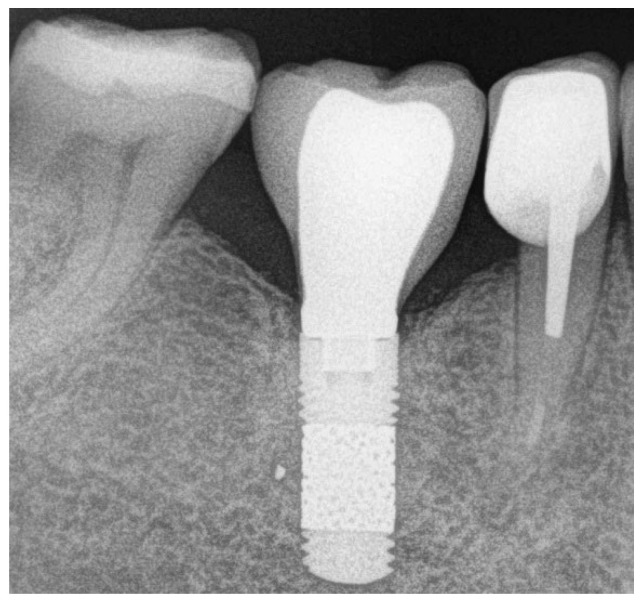
After 2 years of loading, the ISQ values improved to 84 and 83 in the vestibulo-lingual and mesio-distal directions, respectively. The marginal bone level remained stable (as in the previous controls).

Further controls were scheduled at 12 and 24 months of functional loading (Figure 4). At each control, the patients were evaluated clinically and radiographically, and the ISQ was assessed.

**Figure 5:**
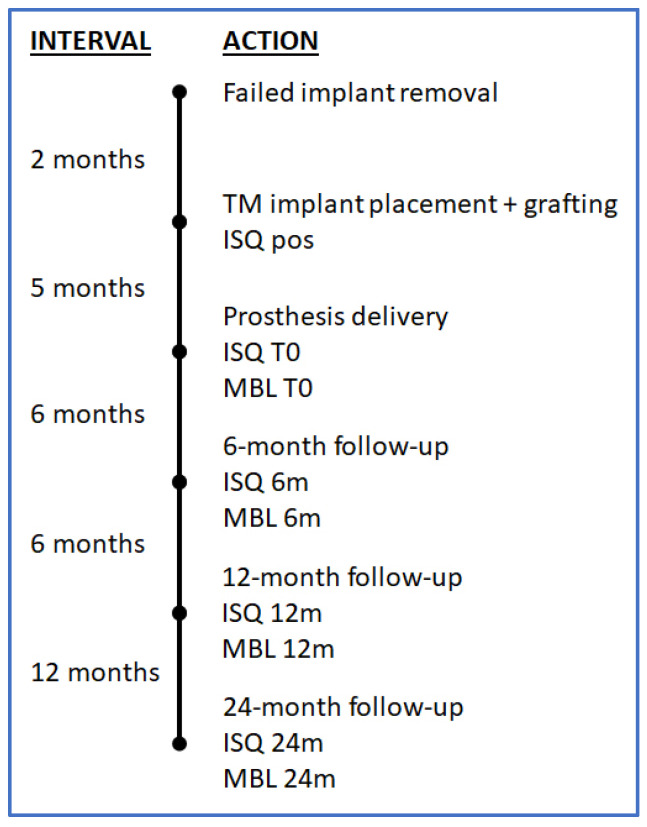
Study timeline and variables measured at each timepoint. ISQ = implant stability quotient; MBL = marginal bone level; pos = at implant positioning; T0 = at baseline.

The study timeline is schematically represented in Figure 5.

### 2.6. Outcome Variables

The outcome variables were as follows:

1. Implant survival, defined as the presence of the implant supporting a prosthetic restoration and surrounded by healthy soft tissues at the time of examination.
2. Implant success, defined as a functional implant with healthy peri-implant tissues and absence of radio transparency around implant sites. According to the current classification [2], three different peri-implant conditions could be identified:
  - Peri-implant health, defined as the absence of clinical signs of inflammation, absence of bleeding and/or suppuration on gentle probing, no increase in probing depth compared to previous examinations, and absence of bone loss beyond crestal bone level changes resulting from initial bone remodeling.
  - Peri-implant mucositis, defined as the presence of bleeding and/or suppuration on gentle probing with or without increasing probing depth compared to previous examinations and the absence of bone loss beyond crestal bone level changes resulting from initial bone remodeling.
  - Peri-implantitis, defined as the presence of bleeding and/or suppuration on gentle probing with or without increasing probing depth compared to previous examinations and the absence of bone loss beyond crestal bone level changes resulting from initial bone remodeling.
3. Bone level changes were evaluated by comparing the measurements at baseline (on the day of prosthesis delivery) with those at follow-up visits. Measurements were obtained with an intraoral radiograph that depicted the vertical distance between the implant shoulder and the most coronal bone contact with the implant surface at mesial and distal sites. All the intraoral radiographs were taken using the parallel technique with an individual tray to ensure reproducibility. Measurements were always performed by the same experienced operator (S.B.) through the software ImageJ version 1.46 (National Institutes of Health, Bethesda, MD, USA), using the known TM implant diameter for calibration. The mesial and distal values were averaged to have a single value per implant.
4. Implant stability was assessed through ISQ values (range 0 to 100), measured using magnetic resonance frequency analysis (RFA, Osstell^TM^ Mentor AB, Gothenburg, Sweden) on the day of implant insertion, at the time of prosthesis delivery, and at each scheduled follow-up control. The latter measurements were performed after unscrewing the prosthetic restoration. The transducer was screwed manually, and ISQ measurements were taken both in the mesio-distal (MD) and bucco-lingual/palatal (BL/BP) dimension by keeping the device perpendicular at a distance between 1 and 3 mm and at least 3 mm away from the soft tissues.
5. Keratinized mucosa width, probing depth, and bleeding on probing were assessed at each follow-up, following the guidelines of the 2017 World Workshop [3]. Pocket probing around implants was carefully conducted using a calibrated periodontal probe and a light force, to not cause disruption of the implant mucosal seal. Bleeding was only scored as present or absent.
6. Any type of biologic and mechanical complication occurring at any time post-surgery.

### 2.7. Sample Size Calculation

The unit of analysis was the case. To achieve statistical significance, an estimated total of 19 cases were required based on the following assumptions: a 95% confidence level (alpha = 5%); a power of 90%; a one-sample method; a minimum of 10% difference in mean ISQ value from restoration to six months of loading; and a standard deviation of 10% (as in a previous study on post-extraction sites [37]). Considering a dropout rate of 5%, it was planned to enroll 20 cases. The estimation was conducted with the online tool Statulator https://statulator.com/SampleSize/ss2PM.html# (accessed on 31 March 2017).

### 2.8. Statistical Analysis

The analysis was carried out using GraphPad Prism (Version 5.03, GraphPad Software, Inc., San Diego, CA, USA). Descriptive statistical analysis was performed. Data were synthesized using the mean values and standard deviation for quantitative variables and absolute or relative frequencies for qualitative variables. The comparisons of the ISQ and bone level changes at the different time frames were made using the paired Student’s *t*-test. The normality of the distributions was assessed using the D’Agostino and Pearson omnibus normality test. Where the normality of distributions was not demonstrated, a non-parametric Wilcoxon matched-pairs test was applied. To test whether there was a relationship among initial defect size, ISQ, and MBL, linear regression analysis was performed. The significance level was set at *p* = 0.05.

## 3. Results

Twenty consecutive cases, referred by other clinics, were enrolled in this study. One of them dropped out due to SARS-CoV-2 infection after five months. The data reported refer to nineteen cases in fourteen patients (seven males and seven females) that completed the follow-up. The mean age was 70.5 ± 11.2 (mean ± standard deviation, SD) years (range 57–89 years). All the patients were regularly monitored at the scheduled times and cooperated in all the phases of this study.

All implants scheduled for removal had a micro-rough surface, in some cases coated with bioactive materials, as confirmed by the post-extraction assessment. Fifteen implants were placed in the mandible and four in the maxilla. Table 1 is a summary of the main patients’ data and ISQ results. The defect depth on the day of explantation averaged 9.07 ± 2.50 mm and 8.96 ± 2.36 mm at the mesial and distal aspects, respectively. The defect width at the crestal level averaged 9.66 ± 1.61 mm and 8.92 ± 1.75 mm in the mesio-distal and bucco-palatal/lingual directions, respectively. The mean insertion torque was 27.1 ± 4.8 Ncm (range 20–35 Ncm).

**Figure 6:**
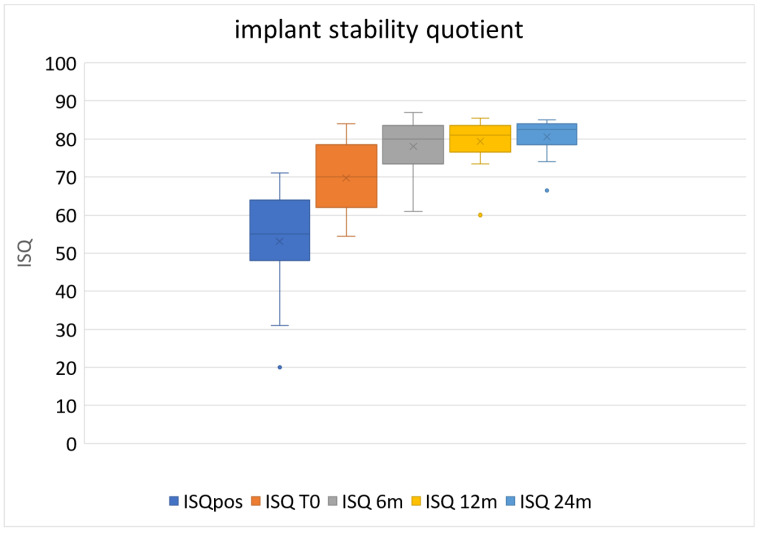
Box-and-whiskers plot showing ISQ pattern up to 24 months of loading. ISQ = implant stability quotient; pos = at implant positioning; T0 = at baseline. The X inside the box represents the mean value.

Figure 6 shows the ISQ values up to 24 months of loading. The ISQ values at the time of implant insertion ranged from 20 to 71 (53.08 ± 13.65).

In many cases, stability could only be achieved in the apical part of the implant. After five months of healing, the ISQ values significantly increased to 69.74 ± 9.01 (*p* < 0.001, paired Student’s *t*-test), with a range from 55 to 84. According to previous studies, such values were considered safe for loading the implants [41,42]. After six months of provisional loading, the mean ISQ value was 78.00 ± 7.29 (range 61–87), which represented a significant increase compared to the previous assessment (*p* < 0.0001). After one year, the mean ISQ value was 79.37 ± 5.89 (range 60–85.5), and after two years, it was 80.55 ± 4.73 (range 66–85). These last two values were not significantly different from the 6-month measurements (see Table 2).

**Figure 7:**
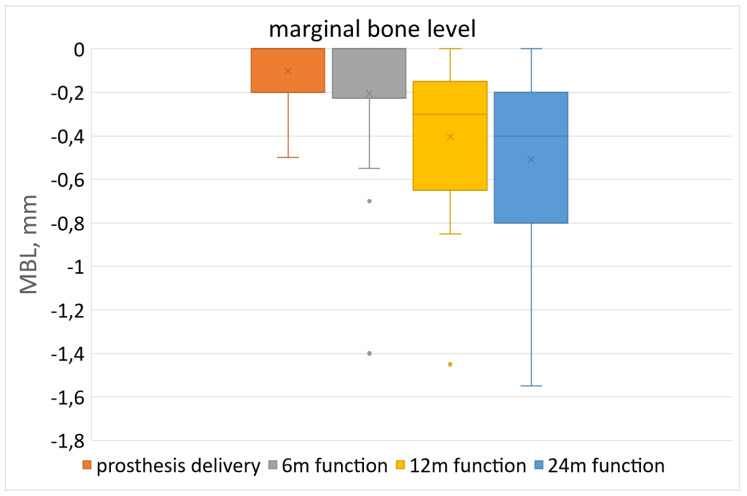
Box-and-whiskers plot showing change in marginal bone level (MBL) pattern up to 24 months of loading. X is the mean value; the horizontal line is the median; upper and lower box limits are the 95% confidence intervals. Dots outside the whiskers represent data outside the 95% CI.

Figure 7 shows the MBL values up to 24 months of loading, with respect to the baseline (T0 = prosthesis delivery).

After the first and the second year of loading, the mean MBL value was 0.40 ± 0.38 mm and 0.51 ± 0.42 mm, respectively. MBL changes at each follow-up are also reported in Table 3. Non-parametric tests were used for comparisons because the MBL distribution was not Gaussian. The only case with more than 1 mm of bone loss corresponded to one patient who had a difficult coverage of the resorbable membrane due to closure with tension. This confirms the importance of an excellent primary closure of the soft tissues around the implants.

After two years of follow-up, the implant survival and success were 100%. No post-surgical biologic nor mechanical complication occurred. Peri-implant soft tissues were healthy at each follow-up, and no sign of peri-implant radiolucency was detected.

No significant correlation was found between the ISQ and the initial defect size in both the horizontal and vertical directions at placement nor at subsequent follow-up. However, it was noted that deeper defects tended to show a larger increase in stability via ISQ values over time, which was comparable to the ISQ value of implants that had good stability at placement. Similarly, no correlation was found between the MBL and the initial defect size for up to two years.

Peri-implant soft tissue thickness after 24 months was ≥3 mm in sixteen out of nineteen cases and between 2 and 3 mm in three cases. No bleeding on probing and no probing depth greater than 2 mm was observed at all the follow-ups.

## 4. Discussion

The design of this study aimed to verify the reliability of implant placement using the same site where implant failure had previously occurred. Based on the encouraging results of our previous study on the insertion in large post-extraction bone defects of natural teeth [42], in this study, we used the same biomaterials and protocol (a patented technique named Overgraft^®^). The achievement and maintenance of implant stability in a previously compromised site, as well as the preservation of peri-implant health, were the main goals of this study. The ISQ was chosen as the variable for sample size calculation, as in our previous studies, because it is objective, quantitative, and may be assessed starting from implant placement, as opposed to other variables, like marginal bone level change, in the present protocol (in fact, the gap around the implant at placement was filled with xenograft) [44].

In this study, surgeries were planned with 2D X-rays along with a clinical evaluation, following the European Association of Osseointegration guidelines for the use of diagnostic imaging. Such guidelines underline that a medical exposure to ionizing radiation must always be justified and result in a net benefit for the patient [45]. Following these guidelines, if the clinician is able to evaluate the bone width and height with 2D imaging, together with a clinical inspection, an implant-supported restoration can be safely performed, reducing the patient dose to ionizing radiation.

When the clinician plans to remove an implant affected by severe peri-implantitis defects, one possible treatment option is immediate replacement by another implant. However, in these cases, it can be challenging to achieve adequate mechanical (primary) stability of the new implant due to the frequent presence of a crater-like circumferential deep defect. An implant with a larger diameter than the one extracted is often used, but this may frequently hamper the preservation of the alveolar crest in the bucco\lingual dimension. In addition, the use of trephine burs for removing the failed implant modifies the shape of the bone defect, making it larger and cylindrical, which results in further difficulty in stabilizing the new implant apically. In these cases, the association of guided bone regeneration (GBR) techniques using autogenous bone and/or bone substitutes and a barrier membrane may be preferred [46]. When the chosen option is a regenerative treatment during the same session as implant extraction, achieving the primary closure may be challenging due to the lack of soft tissues in the emergency area of the abutment. Since these cases often coincide with a reduced amount of keratinized tissue, the management of the membrane cover may become uncomfortable. For the above-mentioned reasons and in analogy with previous studies [40,41,42], further potential complications were avoided in the present study by allowing for the soft tissues to heal and early bone regeneration to take place at the apical part of the defect during the secondary closure after implant removal. For instance, a trabecular metal (TM) implant was inserted concomitantly with a GBR procedure two months after implant removal. The rationale of this procedure is to allow for the formation of an adequate amount of keratinized tissue, which is useful to achieve a good seal that will minimize the potential risk of infection after soft tissue closure [47]. Furthermore, the implant will have a greater chance of achieving primary stability during immediate implant placement. To fill the gap around the implant, the implant was placed first, and then the bone substitute was inserted. The TM implant was inserted before the graft material to avoid any xenograft particles from incorporating into the porous metal trabeculae, thereby hindering or delaying the neo-angiogenesis and new bone formation within the TM structure.

In this study, only cases with peri-implant defects deeper than 50% of the implant length, measured from the residual crest, were considered. Since patients were initially treated in other clinics, the radiograph at the time of insertion or definitive prosthesis delivery was not available. Therefore, the actual amount of peri-implant bone loss in the vertical dimension could not be assessed because it was unknown at which level the implant scheduled for explantation had been placed with respect to the crest. As a result, the measurement of the vertical defect at the time of removal was probably an underestimation of the actual bone loss, and all cases could be considered as advanced peri-implantitis [43].

In our sample, most cases (15 out of 19) were in the mandible, but this was probably only due to the low overall sample size. Though some studies reported a trend towards a higher prevalence of peri-implantitis in subjects with mandibular implants [20,48,49,50], there is no consensus on the influence of implant location as a possible risk factor [51]. A recent review of the literature reported a higher prevalence of peri-implantitis in the maxilla (38.3% out of 5226 implants) as compared to the mandible (28.9% out of 4370 implants) [52].

Given the reduced sample size of the present study, it was difficult to detect if there was a correlation between the ISQ and the implant length and diameter. Also, the correlation between the ISQ and the defect size in both the horizontal and vertical directions at placement was not statistically significant. Also, the ISQ did not correlate with the MBL at any time. One possible explanation for the lack of correlation with the MBL could be the extremely limited values of marginal bone resorption that occurred for up to two years. Despite the non-homogeneous distribution of implants between the jaws, an attempt was made to compare both the ISQ and MBL values in the mandible versus the maxilla, which were not significantly different at any follow-up.

Looking at the trend of ISQ changes over follow-up time, it can be noted that the mean values continue to increase, even after the 6-month follow-up (Figure 6). These increasing values indicate continuous bone remodeling and seem to suggest that TM structures may improve the secondary stability of the implants over time. Notably, a progressive reduction in the standard deviation was observed, indicating that implant stability tends to become more and more consistent with increasing follow-up time. In fact, the ISQ range at the beginning is larger than after six months of loading and tends to decrease over time. The implants with the lowest values at insertion and after five months of healing are the ones that increased more during the first six months of loading, similar to the results of our previous studies using the same protocol on large post-extraction defects, using the same type of implants [40,41,42,53]. The porous trabecular implant structure increases the surface available for bone–implant contact, allowing not only for bone ongrowth but also bone ingrowth into the pores, which enhances implant fixation over time. In contrast, traditional dental implants only allow for bone growth on their surface [54,55,56]. As a result of the three-dimensional bone healing pattern peculiar to TM implants, the term “osseoincorporation” has been coined instead of osseointegration [53]. In addition, the low modulus of elasticity of the TM implants favors a load distribution that reduces the formation of local stress regions [53,54,57,58].

The results on bone resorption are in line with the literature on implants in healed sites, but it is difficult to find comparable literature on bone augmentation cases. This suggests that favorable outcomes can be achieved with the present recovery technique that are similar to those of implants in healed sites, which represents a much less challenging situation. In this study, we aimed at keeping a distance of at least 1.5 mm between the implant surface and the outer buccal and lingual/palatal plate, to minimize peri-implant bone resorption. Previous clinical [59,60], and preclinical [61], studies, as well as a recent systematic review [62], showed that 1.5 to 2 mm of buccal bone wall thickness is a safe distance for limiting marginal bone loss.

The main limitations of the present study are the limited sample size and the absence of a control group, such as a different type of replacement implant or a different treatment approach. Also, according to the original protocol, the unit of analysis was the case not the patient, and some patients had more than one implant treated and analyzed. Once the minimum number of cases planned has been achieved, the enrolment was stopped. In an ideal study, the unit of analysis should be the patient, while this study can be considered as a pragmatic study. In fact, we reported a situation close to the daily clinical practice in which patients can have multiple compromised implants needing treatment. Scarce information was available regarding the explanted implants. For example, it was unknown if implants had been originally positioned at bone level or tissue level and if they had been loaded conventionally or immediately. Early radiographic documentation was also missing. Finally, the same operator performed both the surgical procedure and the clinical and radiological measurements.

Further prospective studies, possibly comparative among different types of implants or different surgical approaches, with a larger sample size and a longer follow-up including different types of defect morphologies are necessary to confirm the present promising data. Nevertheless, the clinical indications for the use of these materials and protocols seem to be well defined.

## 5. Conclusions

A range of solutions has been proposed to address peri-implant disease, with their invasiveness related to the severity of the peri-implant bone loss and peri-implant tissue condition. In the case of large peri-implant bone defects, implant removal and its substitution have been proposed. However, in this clinical scenario, the primary stability is often difficult to achieve due to the lack of hard tissue. In the present study, no failures occurred, no biological complications were recorded, and a good amount of keratinized soft tissue was achieved. Within its limitations, the results of this study suggested that, in combination with the GBR procedure, TM-structured implants can represent a valuable option to recover sites of previous implant failures.

## Figures and Tables

**Table 1:** Patients’ demographics and main individual results.

Case ID	Site	Defect Depth, mm (Mesial)	Defect Depth, mm (Distal)	Defect Width, mm (Mesio-Distal)	Defect Width, mm (Vestibulo-Palatal/-Lingual)	TM Implant Size, mm *	Insertion Torque, Ncm	ISQ at Placement (MD\VL/P)	ISQ 5 m Healing (MD\VL/P)	ISQ 6 m (MD\VL/P)	ISQ 12 m (MD\VL/P)	ISQ 24 m (MD\VL/P)
1	44	7.0	8.1	9.7	6.8	4.1×11.5	30	66\68	84\84	83\81	82\83	82\83
2	44	10.5	10.7	13.3	10.0	4.1×10	20	30\32	71\72	80\83	80\83	81\82
3	46	8.4	9.5	10.7	5.6	4.7×11.5	25	54\53	72\67	74\72	80\82	84\83
4	37	7.1	5.8	7.0	8.1	4.1×11.5	35	65\67	81\80	84\84	83\84	84\85
5	47	6.2	6.4	10.2	9.0	4.7×11.5	30	64\64	80\80	83\84	82\83	83\84
6	14	11.3	10.8	9.9	9.3	3.7×13	20	20\20	62\61	75\78	75\78	76\78
7	46	12.6	13.3	12.1	13.0	4.1×11.5	25	53\52	62\73	82\83	84\84	86\82
8	47	10.8	9.7	12.1	13.0	4.7×11.5	35	71\71	74\74	86\85	85\86	74\74
9	45	15.3	13.5	8.5	8.6	4.1×10	25	54\55	71\70	80\80	80\80	80\82
10	36	10.7	11.0	8.9	9.2	4.1×11.5	30	57\58	79\78	3\82	80\82	80\85
11	45	10.8	11.1	7.6	8.0	3.7×11.5	25	54\56	62\62	73\74	78\78	79\80
12	46	6.4	5.6	9.0	8.4	4.1×10	30	62\64	70\70	80\80	82\83	85\85
13	36	8.6	7.1	10.7	8.7	4.1×11.5	20	46\51	55\55	60\62	73\74	75\76
14	25	7.4	7.8	9.5	9.1	4.1×10	25	58\58	68\69	74\74	76\76	78\79
15	37	6.4	7.4	9.6	9.4	4.7×11.5	30	46\50	56\58	72\79	71\83	83\82
16	27	6.7	7.2	8.2	8.4	3.7×11.5	20	30\32	51\58	61\63	60\60	68\65
17	35	7.2	6.8	7.9	8.2	4.1×10	30	61\60	75\76	84\85	84\85	85\85
18	36	10.5	9.9	9.1	7.5	4.1×10	30	65\64	80\82	87\87	84\84	85\85
19	14	8.5	8.6	9.5	9.1	4.1×11.5	30	42\44	64\64	72\75	74\75	79\79

* = diameter × length; ID = patient identifier; TM = trabecular metal; ISQ = implant stability quotient; m = months; MD = mesio-distal; VL/P = vestibulo-lingual/-palatal.

**Table 2:** Implant stability quotient (ISQ) measurements. The values of each time frame were compared both with baseline (ISQ pos) and with the previous timepoint, using paired Student’s *t*-test.

	ISQ Pos	ISQ T0	ISQ 6 Months	ISQ 12 Months	ISQ 24 Months
Mean value ± SD	53.08 ± 13.65	69.74 ± 9.01	78.00 ± 7.29	79.37 ± 5.89	80.55 ± 4.73
Median (95% CI)	55 (46.50, 59.66)	70 (65.39, 74.08)	80 (74.48, 81.52)	81 (76.53, 82.21)	82.5 (78.27, 82.83)
*p*-value *		1.16 × 10^−6^	2 × 10^−8^	4 × 10^−9^	1 × 10^−8^
*p*-value **		1.16 × 10^−6^	8.53 × 10^−7^	0.12	0.17

* Comparison with respect to the baseline (ISQ pos = implant positioning); ** comparison with respect to the previous timepoint; T0 = prosthesis delivery.

**Table 3:** Marginal bone levels (MBLs). The values of each time frame were compared both with the baseline (T0) and with the previous timepoint, using non-parametric Wilcoxon matched-pairs rank test.

	MBL T0, mm	MBL 6 Months, mm	MBL 12 Months, mm	MBL 24 Months, mm
Mean value ± SD	−0.10 ± 0.16	−0.21 ± 0.37	−0.40 ± 0.38	−0.51 ± 0.42
Median (95% CI)	0 (−0.19, −0.02)	0 (−0.40, −0.01)	−0.3 (−0.60, −0.21)	−0.4 (−0.73, −0.29)
Mean change ± SD		−0.10 ± 0.29	−0.30 ± 0.31	−0.41 ± 0.38
*p*-value *		0.0731	0.0016	0.0013
*p*-value **		0.0731	0.0042	0.014

* Comparison with respect to the baseline (T0 = prosthesis delivery); ** comparison with respect to the previous timepoint. MBL = marginal bone level.

## Data Availability

Data are available upon request to the corresponding author.
